# Phosphorylation Regulates the Endocytic Function of the Yeast Dynamin-Related Protein Vps1

**DOI:** 10.1128/MCB.00833-15

**Published:** 2016-02-16

**Authors:** Iwona I. Smaczynska-de Rooij, Christopher J. Marklew, Ellen G. Allwood, Sarah E. Palmer, Wesley I. Booth, Ritu Mishra, Martin W. Goldberg, Kathryn R. Ayscough

**Affiliations:** a Department of Biomedical Science, University of Sheffield, Sheffield, United Kingdom; b School of Biological and Biomedical Sciences, Durham University, Durham, United Kingdom

## Abstract

The family of dynamin proteins is known to function in many eukaryotic membrane fusion and fission events. The yeast dynamin-related protein Vps1 functions at several stages of membrane trafficking, including Golgi apparatus to endosome and vacuole, peroxisomal fission, and endocytic scission. We have previously shown that in its endocytic role, Vps1 functions with the amphiphysin heterodimer Rvs161/Rvs167 to facilitate scission and release of vesicles. Phosphoproteome studies of Saccharomyces cerevisiae have identified a phosphorylation site in Vps1 at serine 599. In this study, we confirmed this phosphorylation event, and we reveal that, like Rvs167, Vps1 can be phosphorylated by the yeast cyclin-associated kinase Pho85 *in vivo* and *in vitro*. The importance of this posttranslational modification was revealed when mutagenesis of S599 to a phosphomimetic or nonphosphorylatable form caused defects in endocytosis but not in other functions associated with Vps1. Mutation to nonphosphorylatable valine inhibited the Rvs167 interaction, while both S599V and S599D caused defects in vesicle scission, as shown by both live-cell imaging and electron microscopy of endocytic invaginations. Our data support a model in which phosphorylation and dephosphorylation of Vps1 promote distinct interactions and highlight the importance of such regulatory events in facilitating sequential progression of the endocytic process.

## INTRODUCTION

Dynamins are a conserved family of proteins involved in membrane fusion and fission (1–3). While mammalian dynamins are known to be involved in several membrane-trafficking events, the role of dynamin-1 in endocytosis is the best-characterized role of this protein family. Dynamin-1 has been proposed to act largely in a mechanical way to encircle the necks of invaginated endocytic vesicles, facilitating scission of the vesicles during endocytosis (4–6). An additional, regulatory role has also been suggested in which dynamin functions earlier as a molecular switch through its GTPase domain to regulate recruitment or activity of proteins within the endocytic complex (7, 8). Recent studies in live cells have indicated that there is a positive-feedback loop functioning at endocytic sites, with dynamin, actin and N-BAR proteins, such as endophilin and amphiphysin, cooperating to bring about efficient scission of membranes in order to release a vesicle (9, 10). Quantitative fluorescence microscopy of dynamin-2 stoichiometry and dynamics and its functional interdependency with actin have recently been studied. This has revealed that while low levels of dynamin are present early in endocytosis, actin recruitment directly precedes the precise temporal recruitment of further dynamin and that sufficient monomers to generate a single dynamin ring localized to sites prior to scission (11, 12).

Vps1 is a dynamin-like protein and in Saccharomyces cerevisiae is the only dynamin with clear functions in membrane-trafficking pathways (13–17). The other two dynamin-like proteins in S. cerevisiae, Dnm1 and Mgm1, both function in mitochondrial fission/fusion (18, 19), with Dnm1 having an additional function in peroxisomal fission (20–22). Like other dynamins, Vps1 is predicted to have a GTPase domain and a stalk region. However, in common with nonclassical dynamins, it does not have a PH domain, but at an equivalent position in the protein there is an alternative domain referred to as insert B. Despite the absence of a PH domain, we have previously demonstrated that Vps1 is localized to endocytic sites and is able to bind and tubulate liposomes (14). Others have shown interactions between Vps1 and the endocytic adaptor Sla1: defects in uptake of endocytic dyes and disrupted cortical actin (13, 17). Real-time analysis of single endocytic events demonstrates that *vps1* deletion compromises scission events so that retraction to the plasma membrane is commonly observed (14). This phenotype suggests that Vps1 functions in a similar way to Dyn1 or Dyn2 in mammalian endocytosis. In addition, disruption of interactions between Vps1 and its binding partners, the yeast amphiphysin protein Rvs167 and actin, also disrupt endocytic scission (23, 24).

Analysis in yeast has allowed fundamental aspects of endocytic-site assembly and disassembly to be analyzed in detail, and subsequent studies in mammalian cells have demonstrated that key temporal stages of the process are conserved (10, 25, 26). However, major questions remain concerning the progression of endocytosis. How are proteins first recruited and then removed from the site? How are proteins recycled for subsequent endocytic events? It seems likely that posttranslational modifications are central to this process, and many proteins that localize to endocytic sites have been identified in global phosphoproteomic studies (27). However, given that phosphorylation can occur promiscuously, it has been difficult to determine key phosphorylation events that are important for stages of endocytosis to progress. In yeast, later stages of coat disassembly that are proposed to occur concomitantly with scission have been shown to be regulated by Ark1/Prk1 kinases (homologs of mammalian AAK/GAK), and deletion of these kinases prevents disassembly of some proteins, including Pan1 and Sla1, from the endocytic site (28–30). Furthermore, the yeast amphiphysin Rvs167 is phosphorylated by the Pho85 kinase, and mutation of phosphorylation sites compromised its interaction with a regulator of actin polymerization, Las17 (a homolog of WASp) (31). However, in this case, the effects of phosphomutants on endocytic progression were not determined.

In mammalian cells, dynamin has been reported to be phosphorylated by a number of kinases, including Dyrk1, Cdk5, Akt, and glycogen synthase kinase 3 (GSK3) (32–36). Furthermore, phosphorylation-dephosphorylation cycles of dynamin-1 have been shown to be essential for regulating activity-dependent bulk endocytosis during neuronal activity (37).

Global phosphoproteome studies in yeast have identified a phosphorylation site at residue Ser599 in Vps1 (27). This residue is in the insert B region, which we have previously demonstrated also contains a binding site for the SH3 domain of the amphiphysin Rvs167 (23). In this study, we aimed to investigate the function of this phosphorylation event to determine whether the modification plays a role in any or all known Vps1 functions. We confirmed the phosphorylation site using mass spectrometry (MS) and showed that deletion of the Pho85 kinase affects the Vps1 phosphorylation state. We then addressed whether the S599 phosphorylation event plays a regulatory role in cellular activities of Vps1

## MATERIALS AND METHODS

### Materials.

Unless otherwise stated, chemicals were obtained from Sigma-Aldrich (St. Louis, MO). Medium (yeast extract, peptone, and agar) was from Melford Laboratories, Ipswich, Suffolk, United Kingdom, or Sigma (minimal synthetic medium and amino acids). FM4-64 was from Molecular Probes. Phos-tag was from Alpha Laboratories.

### Yeast strains, plasmids, and cell growth.

The yeast strains used in this study are listed in Table 1 and the plasmids in Table 2. Cells were grown with rotary shaking at 30°C in liquid YPD medium (1% yeast extract, 2% Bacto peptone, 2% glucose supplemented with 40 μg/ml adenine) or in synthetic medium (0.67% yeast nitrogen base, 2% glucose) with appropriate supplements. S599V and S599D point mutations in the *VPS1* gene were generated using site-directed mutagenesis (QuikChange Lightning kit; Agilent) with plasmids pKA677, pKA836, pKA695, and pKA850 as the templates. The constructs were then verified by sequencing. Point mutations were introduced into the genome by allele exchange as described previously and verified by sequencing (38). The bimolecular fluorescence complementation (BiFC) assay used strains carrying Venus constructs crossed for coexpression of both N- and C-terminal halves of Venus. Vps1 protein was detected on Western blots of whole-cell extracts using anti-Vps1 antibody (1:2,000 dilution) or antihemagglutinin (anti-HA) antibody (1:1,000 dilution) for strains carrying HA-tagged Vps1. Carboxypeptidase Y (CPY) processing was analyzed in cell extracts as described previously (23). Precleaned CPY antibodies (Chemicon International) were used at 1:100 dilution. Transformations were performed using lithium acetate, as previously described (39). The two-hybrid screen used pJ69-2A (40) and plasmids listed in Table 2.

**TABLE 1:** Yeast strains used in this study

Strain	Genotype	Note
KAY378	*MAT***a** *ura3-52 leu2-3,112 his3-Δ200 lys2-801*(*oc*) *ark1*Δ::*HIS5 prk1*Δ::*URA3*	DDY1563 (28)
KAY446	*MAT***a** *his3*Δ*1 leu2*Δ*0 met15*Δ*0 ura3*Δ*0*	Invitrogen
KAY1539	*MAT***a** *his3*Δ*1 leu2*Δ*0 met15*Δ*0 ura3*Δ*0 pbs2*Δ::*KanMX*	Invitrogen
KAY1542	*MAT***a** *his3*Δ*1 leu2*Δ*0 met150*Δ *ura3*Δ*0 yck1*Δ::*KanMX*	Invitrogen
KAY1544	*MAT***a** *his3*Δ*1 leu2*Δ*0 met15*Δ*0 ura3*Δ*0 yak1*Δ::*KanMX*	Invitrogen
KAY1661	*MAT***a** *his3*Δ*1 leu2*Δ*0 met15*Δ*0 ura3*Δ*0 slt2*Δ::*KanMX*	Invitrogen
KAY1685	*MAT***a** *his3*Δ*1 leu2*Δ*0 met15*Δ*0 ura3*Δ*0 pho85*Δ::*KanMX*	Invitrogen
KAY1668	*MAT***a** *his3 leu2 ura3-52*	LRB758 (66)
KAY1671	*MAT***a** *his3 leu2 ura3-52 yck1*Δ::*URA3 yck2-2^ts^*	LRB756 (65)
KAY1095	*MAT*α *his3Δ1 leu2Δ0 lys2Δ ura3Δ0 vps1*Δ::*KanMX*	E. Hettema
KAY1096	*MAT*α *his3Δ1 leu2Δ0 lys2Δ ura3Δ0 dnm1*Δ::*KanMX vps1*Δ::*HIS5*	E. Hettema
KAY1337	*MAT***a** *Rvs167-GFP*::*HIS3 his3Δ1 leu2Δ0 met15Δ0 ura3Δ0 vps1*Δ::*LEU2*	Ayscough laboratory
KAY1369	*MAT***a** *Sla1-GFP*::*HIS3 his3Δ1 leu2Δ0 met15Δ0 ura3Δ0 vps1*Δ::*LEU2*	Ayscough laboratory
KAY1462	*MAT***a** *his3*-Δ*200 leu2-3,112 ura3-52 trp1-901 gal4Δ gal80Δ LYS2*::*GAL1_UAS_-GAL1_TATA_-HIS3 GAL2_UAS_-GAL2_TATA_-ADE2*	Ayscough laboratory
KAY438	*MAT***a** *his3*-Δ*200 leu2-3,112 ura3-52 trp1-901 gal4Δ gal80Δ LYS2*::*GAL1_UAS_-GAL1_TATA_-HIS3 GAL2_UAS_-GAL2_TATA_-ADE2* (PJ69-2A)	40
KAY302	*MAT*α *ura3-52 leu2-3,112 his3Δ200 trp1-1*	Ayscough laboratory
KAY389	*MAT***a** *ura3-52 leu2-3,112 his3Δ200 trp1-1 lys2-801*	Ayscough laboratory
KAY1756	*MAT***a** *his3*-Δ*200 leu2-3,112 ura3-52 trp1-1 lys2-801 vps1*Δ::*URA3*	Ayscough laboratory
KAY1761	*MAT***a** *his3*-Δ*200 leu2-3,112 ura3-52 trp1-1 lys2-801 vps1S599D*	Ayscough laboratory
KAY1762	*MAT***a** *his3*-Δ*200 leu2-3,112 ura3-52 trp1-1 lys2-801 vps1S599V*	Ayscough laboratory
KAY1621	*MAT*α *his3*-Δ*200 leu2-3,112 ura3-52 trp1-1 lys2-801 RVS167-VC*::*TRP1*	Ayscough laboratory
KAY1832	*MAT***a** *ura3-52 leu2-3,112 his3Δ200 trp1-1 lys2-801 VPS1-VN*::*HIS3*	Ayscough laboratory
KAY1898	*MAT***a** *his3*-Δ*200 leu2-3,112 ura3-52 trp1-1 lys2-801 vps1S599V-VN*::*HIS3*	This study
KAY1899	*MAT***a** *his3*-Δ*200 leu2-3,112 ura3-52 trp1-1 lys2-801 vps1S599D-VN*::*HIS3*	This study
KAY1893	*MAT*α *his3Δ1 leu2Δ0 lys2Δ ura3Δ0 vps1*Δ::*KanMX VPS10-2* × *GFP*	44
KAY1894	KAY389 × KAY1893 cross	This study
KAY1895	KAY1756 × KAY1893 cross	This study
KAY1896	KAY1762 × KAY1893 cross	This study
KAY1897	KAY1761 × KAY1893 cross	This study
KAY1467	*MAT*α *his3Δ1 leu2Δ0 lys2Δ ura3Δ0 vps1*Δ::*KanMx Abp1- mCherry*::*HIS*	Ayscough laboratory

**TABLE 2:** Plasmids used in this study

Plasmid	Description	Origin or reference
pKA552	Gal4BD-Vps1(276-704) *TRP1*	E. Hettema laboratory
pKA167	pGBD-C3::Gal4BD *TRP1*	40
pKA163	pGAD-C2::GalAD *LEU2*	40
pKA730	GalAD-Rvs167 *LEU2*	66
pKA777	Gal4BD-Vps1(276-704) S599V *TRP1*	This study
pKA778	Gal4BD-Vps1(276-704) S599D *TRP1*	This study
pKA544	*URA3*; *CEN* with *PGK*term	
pKA677	pKA544 with *VPS1* (inc 320bp 5′)	This study
pKA781	pKA544 with *vps1* S599V	This study
pKA782	pKA544 with *vps1* S599D	This study
pKA836	YCplac111-VPS1-GFP own promoter; *LEU CEN*	14
pKA859	YepLac112-*RVS167 TRP1* 2μ	Hicke Laboratory
pKA902	pKA836 with *vps1 S599V*	This study
pKA903	pKA836 with *vps1 S599D*	This study
pKA695	YCplac111-*pTPI1* 3×*HA-VPS1 LEU CEN*	This study
pKA904	pKA695 with *vps1 S599V*	This study
pKA905	pKA695 with *vps1 S599D*	This study
pKA850	His-tagged Vps1 wild type	14
pKA971	pKA850 with *vps1 S599V*	This study
pKA972	pKA850 with *vps1 S599D*	This study
pKA1051	pGal1/10-GST-GST-PHO85	Open BioSystems

Epifluorescence microscopy, uptake of FM4-64, time-lapse live-cell imaging of green fluorescent protein (GFP)- or mCherry-tagged proteins, and all image processing were performed as described previously (23). Time-lapse live-cell images of Rvs167-GFP were acquired using OMX DeltaVision V4 and a 60× USPLAPO (numerical aperture, 1.42) objective with refractive index 0.1514 immersion oil (Cargille). Samples were illuminated using Insight Solid State Illuminator (10%), and images were taken simultaneously on separate scientific complementary metal oxide semiconductor (sCMOS) cameras (70-ms exposure). Six 200-nm sections were acquired every 510 ms (180 time points). The stacks were then deconvolved and processed, using SoftWorx, to produce a movie composed of maximum intensity projections at each time point. The Rvs167-GFP lifetime was analyzed from those projections.

For electron microscopy analysis, yeast cells were grown to mid-exponential phase at 30°C and harvested by syringe filtration. The concentrated cells were transferred to a Leica flat specimen carrier and frozen in a Leica Empact high-pressure freezer. Freeze substitution and fixation were carried out in a Leica AFS freeze substitution unit. Frozen specimens were fixed using fixative (0.1% uranyl acetate, 2.5% glutaraldehyde, 5% H_2_O in acetone) at −90°C for 48 h and then warmed to −25°C for 12 h. The fixative was removed by three 15-min washes in acetone at −25°C. Samples were infiltrated with Lowicryl HM 20 resin (Polysciences), and the resin was polymerized by UV irradiation. The blocks were cut into 40- to 60-nm-thick sections using a Diatome Ultrasonic diamond knife on a Leica EM UC6 ultramicrotome (Leica Microsystems). The sections were stained with 1% aqueous uranyl acetate for 10 min, washed 20 times with water and stained with Reynold's lead citrate for 10 min, washed again, and air dried. The endocytic invaginations on the plasma membranes of these yeast cells were viewed at 100 kV in a Hitachi H7600 transmission electron microscope (TEM). The lengths of identified invaginations were measured using ImageJ software.

Purification of His-tagged recombinant Vps1, as well as analysis of Vps1 rings by electron microscopy, was performed as described previously (14). Recombinant Vps1 was used at 2.5 μM for incubation with PIP strips and PIP arrays according to the manufacturer's instructions (Echelon). Binding was detected using anti-His antibodies (AbCam).

### Purification of HA-Vps1 for mass spectrometry analysis.

Exponentially grown cells of the *vps1*Δ strain, carrying 3×HA-Vps1 under the control of the *TPI1* promoter (1,000 optical density at 600 nm [OD_600_] units), were centrifuged (3,000 rpm; 3 min; 4°C), and the pellet was resuspended in 2 ml lysis buffer with protease and phosphatase inhibitors (50 mM Tris-HCl, pH 7.5, 100 mM NaCl, 0.1% Triton X-100, 0.1% β-mercaptoethanol, 1 mM EGTA, 2.5 mM EDTA, 1 mM phenylmethylsulfonyl fluoride [PMSF], protease inhibitor cocktail without EDTA [Roche], 10 μM leupeptin, 1 μM pepstatin A, PhosStop cocktail [Roche], 1 mM NaF, 0.5 mM Na_3_VO_4_). The cell suspension was dripped directly into liquid nitrogen and broken by mortar and pestle grinding to obtain a fine powder. The powder was left to thaw on ice for 15 min and stirred into solution by addition of lysis buffer (plus glycerol; 2% final concentration) to 5 ml. The lysate was centrifuged (10,000 rpm; 5 min; 4°C), and the supernatant was precleared with 20 μl of anti-mouse IgG–agarose at 4°C for 1 h, followed by incubation with 50 μl slurry of EZview red anti-HA affinity gel (Sigma-Aldrich) at 4°C for 2 h. The HA-Vps1-bound gel was washed three times with lysis buffer before addition of 50 μl 2× protein-loading buffer and heating at 100°C for 5 min. Samples were run on a Criterion TGX precast gel (AnykD; Bio-Rad) and stained with SimplyBlue SafeStain (Invitrogen), and the HA-Vps1 band was excised for MS analysis. The excised gel pieces containing an HA-Vps1 band were washed, reduced, alkylated, and in-gel tryptic digested. MS analysis was performed by the biOmics Facility, University of Sheffield, Sheffield, United Kingdom.

### Affinity purification of GST-Pho85.

To prepare cell lysates of the *vps1*Δ strain carrying glutathione *S*-transferase (GST)–Pho85 under the control of the GAL1/10 promoter, 1,000 OD_600_ units of cells grown in synthetic medium with 2% galactose were centrifuged at 3,000 rpm for 3 min at 4°C, and the pellet was resuspended in 10 ml of ice-cold lysis buffer containing protease and phosphatase inhibitors (as described above) with added glycerol to a 2% final concentration. Cell disruption was performed using a French press, and the supernatant after centrifugation was incubated with a 100-μl slurry of glutathione-Sepharose 4B beads (GE Healthcare) at 4°C for 16 h. The GST-Pho85-bound beads were washed with lysis buffer (with inhibitors) and then 3 times with kinase buffer (50 mM Tris-HCl, pH 7.5, 10 mM MgCl_2_, 1 mM EGTA, 0.1% Tween 20, 1 mM PMSF, protease inhibitors) before resuspension in 150 μl of kinase buffer with 20 mM reduced glutathione (Sigma) and incubation for 1 h at 4°C. The beads were then pelleted, and 50 μl of supernatant was used for the *in vitro* kinase assay, as described below.

### HA-Vps1 overexpression and immunoprecipitation, alkaline phosphatase (CIAP) treatment, and *in vitro* kinase assay.

Lysates of the *vps1*Δ strain, carrying 3×HA-Vps1 under the control of the *TPI1* promoter, were generated as for mass spectrometry, except cell disruption was performed using a prechilled French press (10,000-lb/in^2^ pressure). Following centrifugation, the supernatant lysate was incubated with 60 μl slurry of EZview red anti-HA affinity gel (Sigma-Aldrich) at 4°C for 16 h before the gel was washed with lysis buffer (with protease and phosphatase inhibitors as described above). One-third of the precipitate was resuspended in 25 μl of 2× protein-loading buffer and boiled for 5 min. For calf intestinal alkaline phosphatase (CIAP) treatment (New England BioLabs), the remaining immunoprecipitate was resuspended in 50 μl of lysis buffer with 10 mM MgCl_2_ and 25 U of CIAP and incubated at 37°C for 2 h with gentle shaking. Half of this sample was then washed with lysis buffer (with added protease inhibitors), and the CIAP reaction was stopped by addition of 25 μl of 2× protein-loading buffer and boiling for 5 min. The remaining half of the sample was washed three times with lysis buffer (with protease and phosphatase inhibitors to stop the CIAP reaction) and then three times with kinase buffer with protease and phosphatase inhibitors. Beads were resuspended in 50 μl of solution containing affinity-purified GST-Pho85 with 70 μM ATP and incubated at 37°C for 45 min. The beads were washed 3 times with kinase buffer, resuspended in 25 μl of 2× protein-loading buffer, and boiled for 5 min. Samples were separated using a 7.5% SDS gel containing 20 μM Phos-tag and 40 μM MnCl_2_. After electrophoresis, the gel was soaked with gentle agitation for 10 min, first in transfer buffer with 1 mM EDTA and then without EDTA to remove manganese ions. For Western blot analysis, the membrane was first incubated with blocking solution (2% bovine serum albumin) at room temperature for 1 h and then at 4°C overnight with a 1:1,000-diluted primary antibody (rat monoclonal anti-HA; Roche).

## RESULTS

### Vps1 phosphorylation *in vivo* and *in vitro*.

**FIG 1:**
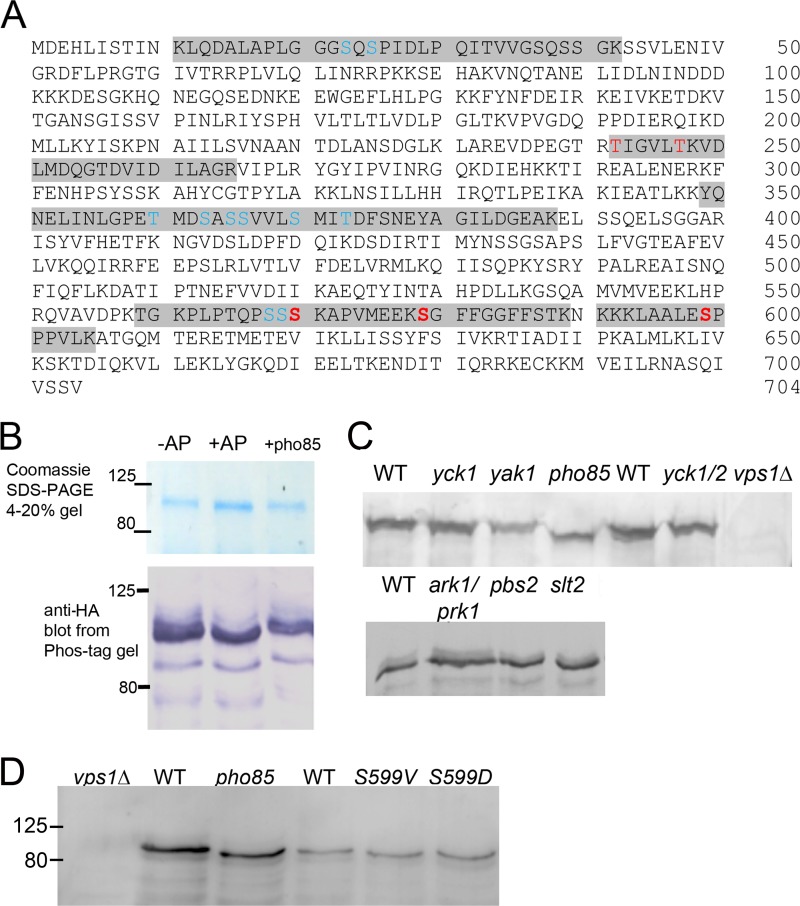
Vps1 phosphorylation *in vivo* and *in vitro*. (A) Vps1-HA was immunoprecipitated from log-phase cells and subjected to analysis by mass spectrometry as described in the text. Peptides carrying phosphorylated residues are highlighted. High-confidence sites are shown in red (high-confidence identity, PEP < 0.01; high-confidence localization, 1% false-localization rate). Other sites (blue) were identified either with medium confidence (PEP > 0.01) but with high-confidence localization or with high confidence identity (PEP < 0.01) but low-confidence localization (>1% false-localization rate). (B) Immunoprecipitated Vps1 was incubated in the presence (+) or absence (−) of calf intestinal AP. Following AP treatment, a fraction of the HA-tagged Vps1 was incubated in the presence of GST-Pho85, as described in the text. Extracts were separated both on an Any Kd gradient gel or on SDS-7.5% PAGE containing 20 μM Phos-tag additive, followed by blotting and probing for Vps1 with anti-HA antibodies. (C) Whole-cell extracts were made from strains with deletions of kinase genes. Extracts were separated on gels (10% gel; 100 μM Phos-tag) and blotted as described in the text. The blots were probed with anti-Vps1 antibodies. WT, wild type. (D) The Pho85 consensus site at Ser599 was mutagenized to valine or aspartate. Extracts from cells expressing these were made, separated, blotted, and probed with antibody. The *pho85* deletion and *vps1* mutants were not isogenic, so different wild-type strains were used for extracts. Numbers indicate the sizes of molecular weight markers.

To verify the global phosphoproteome studies, mass spectrometry was performed on HA-tagged Vps1 that had been isolated on beads from wild-type yeast extracts growing in the exponential growth phase. Five high-confidence sites were identified (high-confidence identity, posterior error probability [PEP], <0.01; high-confidence localization, 1% false-localization rate) at Ser599, Ser579, Ser 570, Thr242, and Thr247 (shown in red in Fig. 1A). In addition, other sites were identified, either with medium-confidence identity (PEP > 0.01) but with high-confidence localization or with high-confidence identity (PEP < 0.01) but low-confidence localization (>1% false-localization rate). The positions of these are marked in blue in Fig. 1A.

To further investigate phosphorylation in our yeast strains, Vps1-HA was overexpressed and immunoprecipitated. One part of the immunoprecipitate was incubated with alkaline phosphatase to remove phosphate groups. A small size shift could be detected on a standard SDS-PAGE gradient gel (Fig. 1B, top, middle lane). However, because of the difficulties in detecting small size shifts in relatively large proteins (>80 kDa), the gel additive Phos-tag was used. Phos-tag is an additive that is able to bind to phosphate groups, increasing their mass and thus making changes in the phosphorylated state easier to detect. The immunoprecipitates were run on a gel and blotted. As shown in Fig. 1B, bottom, Vps1-HA incubated with alkaline phosphatase (+AP) has fewer bands and slightly increased mobility in the gel, indicating removal of phosphate groups from the protein.

In order to investigate the role of possible kinases in this phosphorylation event, whole-cell extracts were made from cells in which genes for specific kinases with a reported link to endocytosis had been deleted. Vps1 was not tagged in these experiments. The mobility of bands was observed to determine possible changes in phosphorylation. As shown in Fig. 1C, deletion of *yak1*, *pbs2*, *slt2*, *yck1*, or *yck2* as a single mutation did not show a change in mobility of Vps1. Yck1 and Yck2 single deletions have little reported effect in cells, but the double deletion is lethal; thus, a strain with a deletion of *yck1* and a temperature-sensitive allele of *yck2*, as well as an isogenic parental wild-type strain (different from the wild types of the above-mentioned strains) were also investigated after 4 h growth at the restrictive temperature. Again, no shift was observed for Vps1. Similarly, a double deletion of *ark1* and *prk1* kinases did not show a mobility shift. In contrast, deletion of the kinase Pho85 gene caused a marked band shift, indicating a reduction in phosphorylation and identifying Pho85 as a kinase capable of phosphorylating Vps1. Interestingly, Pho85 has also been reported to phosphorylate Rvs167, which we have previously shown to interact with Vps1. To further confirm Pho85 as a Vps1 kinase, the phosphatase-treated immunoprecipitated Vps1 (described above) was incubated with GST-Pho85 purified from yeast. Following incubation, proteins were run on a gel and blotted, and as shown in Fig. 1B (right lane), there was a clear mobility shift, indicating that Pho85 is able to phosphorylate Vps1.

Analysis of consensus phosphorylation sites has demonstrated that Pho85 has a strong proline-directed site preference, with a serine being phosphorylated when directly N terminal of a proline residue. Of the phosphorylation sites detected by mass spectrometry, a single site, Ser599, matches this consensus. S599 was mutated to valine (nonphosphorylatable) and to aspartate (phosphomimetic), and whole-cell extracts were made to determine whether this mutation altered the mobility of the protein in a gel. As shown in Fig. 1D, both the Vps1 S599V and S599D mutant proteins ran at the same size as the protein in the *pho85* deletion strain and at a smaller size than the wild-type protein (from both parental background strains), indicating that these mutant proteins have reduced phosphorylation.

### Vps1 phosphomutants function normally in nonendocytic functions.

**FIG 2:**
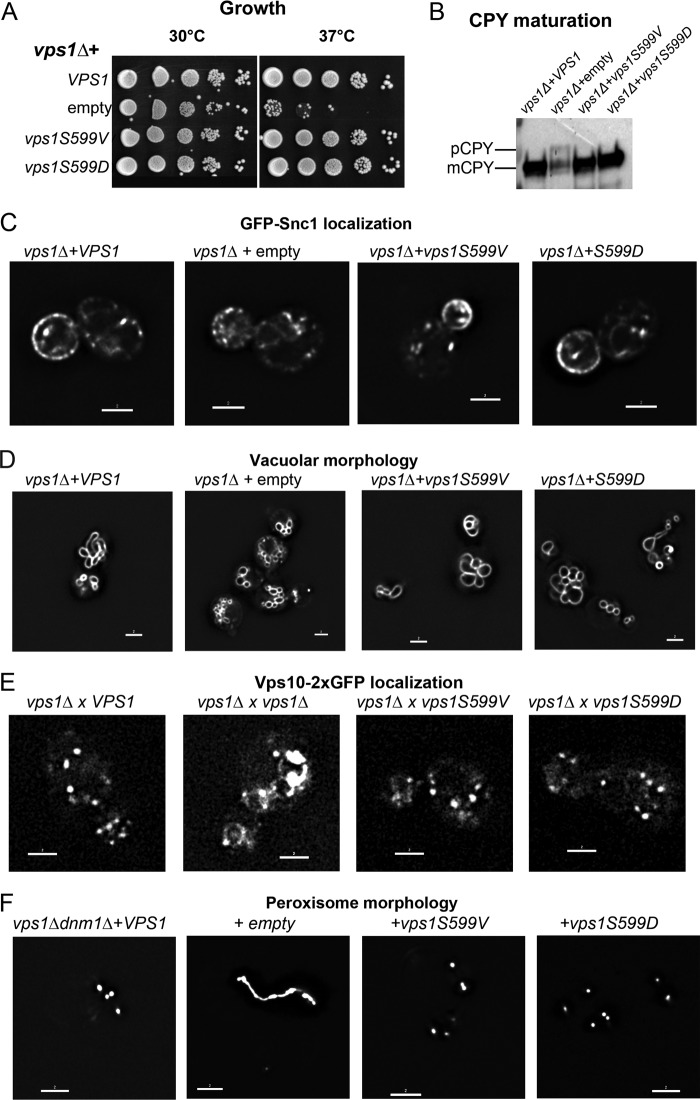
Vps1 S599 mutants can perform Vps1-requiring functions in cells. (A) Cells expressing wild-type *VPS1*, a *vps1* deletion, or the Vps1 S599V and S599D mutants were assessed for growth at 30°C and 37°C. As shown, both the S599V and S599D mutants rescue the temperature sensitivity associated with the complete deletion. (B) Carboxypeptidase Y maturation was assessed in cells, and only the deletion strain was observed to have reduced levels and to show the presence of the higher-molecular-weight immature CPY band. (C) Localization of the GFP-Snc1 reporter that cycles from Golgi apparatus to plasma membrane to endosomes was analyzed for changes in distribution. (D) Vacuolar morphology was assessed following labeling with the lipophilic dye FM4-64. (E) A Vps10-2×GFP *vps1*Δ strain was crossed with strains expressing the wild type or *vps1* mutants to determine whether the mutants rescue the retrograde trafficking defect associated with *vps1* deletion. (F) Peroxisome morphology was assessed in cells carrying a *vps1 dnm1* double deletion with *VPS1* and the *vps1* mutants reintroduced.

Because Pho85 appears to phosphorylate S599 *in vivo* and because it has previously been linked with Rvs167 function, we focused analysis on the function of the serine 599 residue. In order to determine the *in vivo* consequences of loss of Vps1 phosphorylation, S599V and S599D *vps1* mutants were generated and expressed in cells lacking endogenous *vps1* expression. As shown in Fig. 2A, while *vps1* deletion cells exhibit only slow growth at 37°C, both mutants are able to rescue growth to wild-type levels.

Vps1 has been shown to function in Golgi apparatus-to-vacuole trafficking of the enzyme CPY (41, 42). This transport can be followed by analysis of modified forms of the protein so that in wild-type cells, only the mature vacuolar form (mCPY) is observed, while in mutants with defective transport, there is an accumulation of a higher-mass precursor form of the protein (pCPY). In these mutants, CPY is also secreted from cells, so reduced levels of protein are observed. In the absence of Vps1, pCPY is observed, while both mutant strains are indistinguishable from cells with wild-type *VPS1* (Fig. 2B).

Vps1 has also been shown to function in endosomal recycling such that, in its absence, a reporter construct, GFP-Snc1, is found predominantly as small punctae in cells, with only 10% ± 2% of cells showing any polarized plasma membrane staining. In wild-type cells, this reporter is found largely polarized to the bud plasma membranes of growing buds of cells, as well as in internal structures that are generally larger and more intensely stained than in the *vps1* deletion strain (97% ± 2% of cells) (23, 43). In both S599 mutants, the GFP-Snc1 localization resembles that observed in wild-type cells (S599V, 96% ± 2%, and S599D, 95% ± 1% bud-polarized plasma membrane staining; *n* = 3 experiments), suggesting the mutant proteins expressed are capable of supporting the endosomal-recycling function of Vps1 (Fig. 2C).

One of the original phenotypes associated with *vps1* loss of function was defective vacuolar morphology. Rather than 3 to 10 evenly sized vacuoles that could elongate along the mother bud axis during inheritance, vacuoles were more heterogeneous and often appeared to surround a larger structure, which was considered likely to be a vacuole but which was not labeled (41). This is the class F category of vacuolar-protein-sorting mutants. As shown in Fig. 2D, the S599 mutants have a vacuole morphology similar to that of the wild type, while the *vps1* deletion strain has the classic class F phenotype, with smaller vacuoles surrounding a larger, unlabeled structure. More recently, a clear role for Vps1 in fission of retrograde carriers from endosomes has been reported. In the *vps1* deletion strain, a Vps10-2×GFP fusion protein undergoes a prominent shift from Golgi apparatus and endosome localization to the vacuolar membrane, similar to the phenotype observed for retromer-deficient cells (44). As shown in Fig. 2E, expression of wild-type *VPS1* and both Vps1 phosphomutants rescues this phenotype, indicating that phosphorylation of this residue does not functionally affect the fission event.

Finally, Vps1 has also been shown to function in peroxisome fission, so that in the absence of both *vps1* and *dnm1*, there is a single large peroxisomal structure (20). Reexpression of *VPS1* rescues this fission defect and restores peroxisome numbers. Both S599 mutants are also capable of supporting peroxisome fission (Fig. 2F).

Taken together, the data indicate that mutation of S599 does not compromise Vps1 in its functions in membrane fission steps at sites away from the plasma membrane.

### Vps1 phosphomutants have defects in endocytic scission.

**FIG 3:**
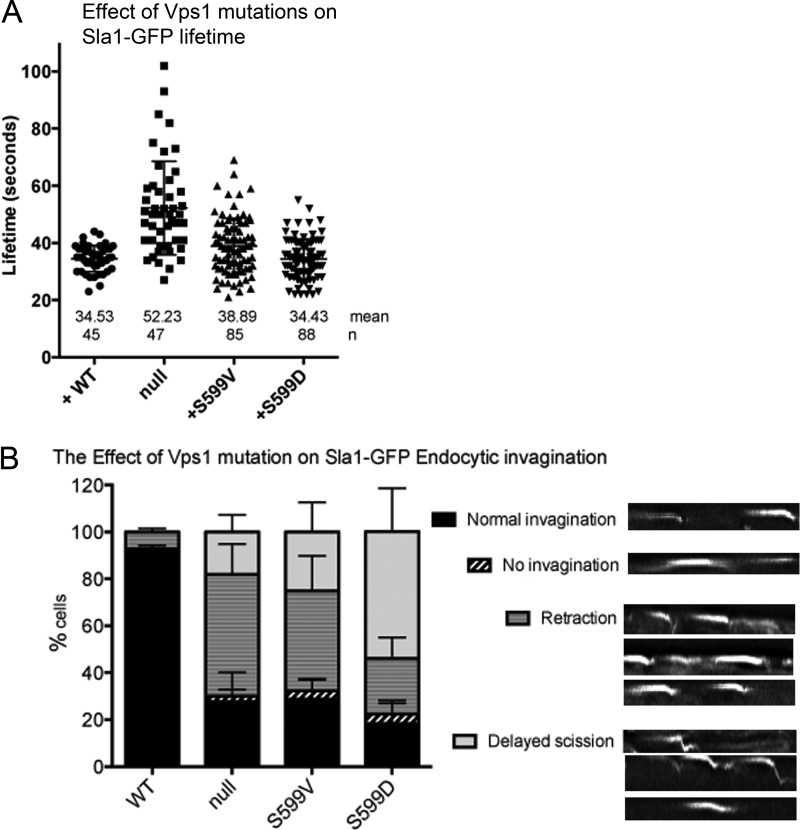
Effects of Vps1 mutations on behavior of an early endocytic marker, Sla1-GFP. (A) The lifetimes of Sla1-GFP at endocytic sites were measured. Mean lifetimes and numbers of patches counted are shown. Only the *vps1* deletion strain showed a lifetime significantly different from that of the wild-type strains. (B) The behavior of Sla1-GFP patches was assessed and categorized as indicated. Patches were considered to endocytose normally, show no invagination, show retraction, or show invagination followed by a delay in the invaginated state. Examples of kymographs depicting these behaviors are shown. Error bars indicate standard deviations.

Previously, we have generated Vps1 mutants that are disrupted only in their endocytic function while rescuing other Vps1 functions, including those mentioned here, such as growth at 37°C and peroxisomal fission. These mutants include Vps1 P564A, which is unable to bind the amphiphysin Rvs167, and Vps1 RR457,458EE, which is unable to bundle actin filaments (23, 24). To determine whether the S599V or S599D mutations were compromised in endocytosis, the fates of individual reporters were assayed. Sla1 is a protein that binds to cargo and clathrin and also serves as a link, via Las17 and Sla2, to the actin cytoskeleton (45–47). Sla1-GFP behavior was monitored in cells expressing *vps1* mutants. The mutant proteins did not cause significant changes in the lifetime of the reporter compared to cells expressing the wild-type Vps1. However, the behavior of the reporter at the plasma membrane during this time was altered in the mutants. Using data from kymographs, patches were analyzed as to whether they exhibited normal invagination, retraction, delayed scission, or no invagination (examples of these behaviors are depicted). As shown in Fig. 3B, while 90% of the patches show normal invagination in wild-type cells, this is reduced to less than 30% in the *vps1* deletion and both mutant strains. Further analysis revealed that in the *vps1*-null strain and Vps1 S599V mutant, the predominant behavior was retraction, when the patch began to show some invagination and then returned toward the plasma membrane before protein disassembly. The Vps1 S599D mutant had a slightly different phenotype, with the predominant behavior being delayed scission when the patch moved a normal or extended distance but then stayed in this invaginated state before Sla1 was disassembled.

### Vps1 phosphomutants still retain the ability to localize to endocytic sites.

**FIG 4:**
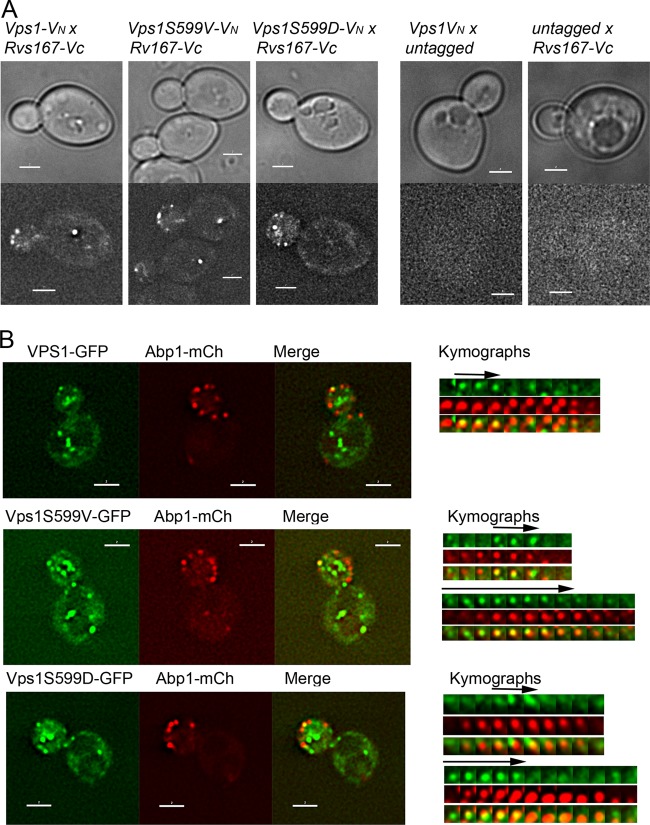
Localization of Vps1 phosphomutants to endocytic sites. (A) Bimolecular fluorescence assay with Vps1 tagged with the N terminus of Venus (V_N_) and Rvs167 with the C terminus of Venus (V_C_). The wild type and both Vps1 phosphomutants were analyzed. Controls on the right demonstrate absence of signal on expression of either half of the Venus construct. (B) Localization of Vps1 to endocytic sites was determined by colocalization of Vps1-GFP with Abp1-mCherry. On the right of the whole-cell images are sequential images from time-lapse movies of individual patches. The arrows depict Vps1-GFP colocalization with Abp1-mCherry.

Given that endocytic behavior of patches is compromised in the mutant cells, it was relevant to determine whether these phosphomutant Vps1 proteins are able to localize to appropriate cortical sites. We and others have noted that GFP-tagged Vps1 is not fully functional, and we proposed that a likely reason for this is that Vps1, like other dynamins, forms a ring structure, which is unlikely to accommodate 100% tagged protein (38, 44). Thus, while a fluorescent tag can be used to indicate localization of Vps1, the lifetime and behavior of the protein are most likely compromised. To determine whether the phosphomutants are able to localize to appropriate cortical sites, we first used a bimolecular-fluorescence approach in which Vps1 tagged with one half (V_N_) of a split yellow fluorescent protein (YFP) Venus tag could localize in the proximity of Rvs167 tagged with the complementary half of the tag (V_C_). As shown in Fig. 4A, while neither tag alone could elicit a signal, in combination, both the wild type and Vps1 mutants are able to localize to endocytic sites so that the V_N_ and V_C_ tags are in close enough proximity to allow a signal to be generated. A second approach analyzed colocalization of Vps1-GFP and Abp1-mCherry. In wild-type cells, brief periods of colocalization could be observed with lifetimes of 6 to 8 s, similar to those previously reported (14). In all cases, Abp1-mCherry was observed to localize to the sites prior to Vps1-GFP. Intriguingly, in the case of the mutants, two classes of localization were observed. In about 40% of cases, the spots were as in the wild-type case, with Abp1-mCherry arriving first followed by a short colocalization of about 6 to 8 s with Vps1-GFP. However, in about 60% of cases with both mutants, Vps1-GFP was observed at sites prior to Abp1-mCherry. In these cases, the period of colocalization was prolonged (10 to 14 s). While the effect of the tag on functionality needs to be considered, these data suggest that the presence of mutations at S599 somehow compromises the turnover of Vps1 at endocytic sites.

### Vps1 phospho-site mutations affect the interaction with the amphiphysin Rvs167.

**FIG 5:**
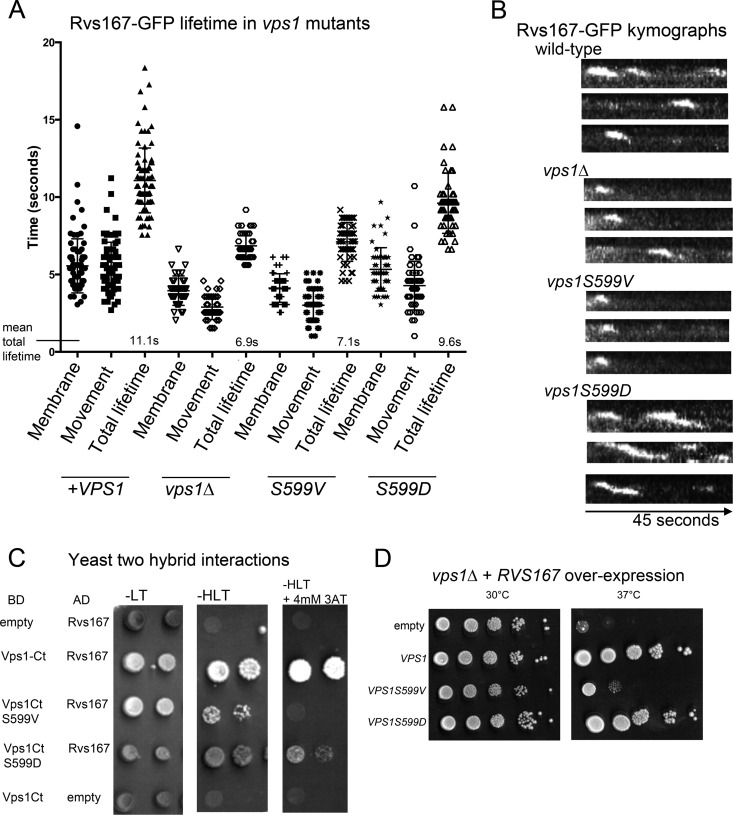
Effect of Vps1 S599 mutations on amphiphysins. (A) The lifetimes of the amphiphysin Rvs167-GFP at endocytic sites were measured for each of the strains indicated and categorized as two stages: either the lifetime at the membrane or time in invagination/movement. Total mean lifetimes are indicated. Error bars indicate standard deviations. (B) Representative kymographs of Rvs167-GFP in each of the strains. The time course for kymographs was 45 s. (C) Yeast two-hybrid analysis was used to investigate interactions between the Vps1 C-terminal region (amino acids 276 to 704) as a Gal4 binding domain fusion and Rvs167 fused to the Gal4 activation domain. Controls with empty plasmids are shown. −LT, medium lacking leucine and tryptophan; −HLT, medium lacking histidine, leucine, and tryptophan. (D) Rvs167 overexpression on a 2μ inhibits cell growth at 37°C. The effect of cooverexpression of Vps1 on this phenotype was investigated. Both wild-type Vps1 and Vps1 S599D were able to rescue the temperature sensitivity.

Given that an interaction between Vps1 and the amphiphysin Rvs167 has been previously reported and that deletion of *rvs167* also causes retraction phenotypes, it was also relevant to determine whether the Rvs167 lifetime at the site is altered by the mutations. In this study, rather than simply recording the lifetime in a single plane, an OMX microscope setup was used that allowed rapid recording of full z-stacks to allow any movement out of a single focal plane to be followed. The lifetimes of Rvs167 both at the plasma membrane (Fig. 5A, Membrane) and during the invagination (Fig. 5A, Movement) were differentiated in order to determine whether distinct stages of endocytosis were affected. As shown in Fig. 5A and in kymograph form in Fig. 5B, in the absence of Vps1, the lifetime of Rvs167-GFP is reduced compared to that in cells in which wild-type *VPS1* is expressed (23). The stage most affected is the invagination stage, with the time spent in this stage reduced from 5.5 s (*VPS1*) to 2.9 s (*vps1*Δ). Interestingly, analysis of the Rvs167-GFP lifetime in the mutants indicated that in the nonphosphorylatable S599V mutant, Rvs167-GFP had a markedly shorter lifetime in both stages, similar to the null strain, while in the phosphomimetic S599D mutant, the Rvs167 lifetime was more similar to that observed in cells expressing wild-type *VPS1*. The effect of the S599V mutation on shortening the lifetime is clear from kymographs. This analysis also shows the delayed scission phenotype for the S599D mutant that was also noted in the Sla1-GFP analysis.

The different lifetimes of Rvs167-GFP in the two mutants suggested that the mutants might be differentially affected in their physical interaction with Rvs167. Previously, an interaction between Rvs167 and Vps1 was shown both using yeast two-hybrid analysis and with purified proteins (23). Here, we used yeast two-hybrid analysis to determine whether the serine 599 mutations affected the Rvs167 interaction. As shown in Fig. 5C, the S599V mutation is severely compromised in its interaction with Rvs167, while the S599D interaction appears more modestly reduced. The inhibited interaction of S599V with Rvs167 might therefore underlie the reduced timing of Rvs167 localization to endocytic sites. Further evidence for the effect of the mutations on an interaction between Rvs167 and Vps1 *in vivo* was shown using genetic approaches. In the absence of *vps1*, Rvs167 overexpression was detrimental to the cells at elevated temperatures (Fig. 5D) (48). This lethality could be rescued by cooverexpression of Vps1, indicating that the interaction renders excess Rvs167 nontoxic to the cell. Overexpression of the Vps1 S599D mutant is also able to rescue Rvs167 overexpression-induced lethality to an extent similar to that with wild-type Vps1, supporting the idea that an interaction is still able to occur. In contrast, overexpression of Vps1 S599V is not able to rescue lethality. Taken together, the data support the idea that phosphorylation of S599 is able to regulate the interaction between Vps1 and Rvs167. This lack of Vps1-Rvs167 interaction in the Vps1 S599V mutant cells confers a phenotype very similar to that previously reported when the PXXP SH3 binding site (P564A) in Vps1 was mutated (23).

### Vps1 phosphomutants have extended endocytic invaginations.

**FIG 6:**
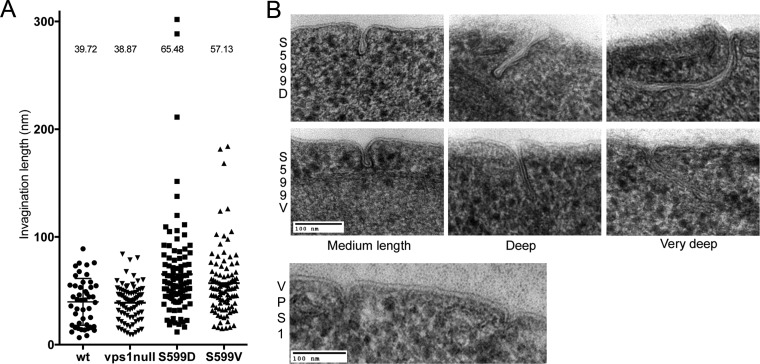
Effect of S599 mutation on ultrastructure of plasma membrane invaginations. (A) Cells expressing the wild type or *vps1* mutations were high-pressure frozen and processed for electron microscopy as described in the text. The lengths of invaginations observed in cells were measured. Each spot represents one measurement, and means are indicated. Both S599D and S599V cause a statistically significant increase in invagination length. Error bars indicate standard deviations. (B) Images of wild-type invaginations, as well as average and long invaginations for the S599V and S599D mutants.

Although kymographs indicate that scission might be inhibited or delayed in the Vps1 S599 mutants, it is not possible to visualize these defects directly using fluorescence microscopy. To determine whether the S599 mutations affected the ultrastructure of endocytic invaginations, cells were fixed by high-pressure freezing followed by freeze substitution. Following staining, sections were analyzed for the presence of plasma membrane invaginations. Where membrane profiles could be followed for the full length of the invagination, the length of the invagination was recorded. As shown in Fig. 6, expression of *vps1* carrying mutations in the S599 site resulted in a significant increase in the average invagination length. Cells with wild-type *VPS1* had a mean invagination length of 37 nm compared to 65.5 nm in Vps1 S599D cells and 57.1 nm in Vps1 S599V mutants. These increases were considered significant in a one-way analysis of variance (ANOVA) using Dunn's multiple-comparison test. Images of invaginations in the wild-type and mutant strains are shown. The data confirm those obtained from fluorescence microscopy, indicating that both phosphorylation and dephosphorylation are likely to be important for appropriate Vps1 functioning during endocytosis.

### Analysis of purified Vps1 S599 mutants.

**FIG 7:**
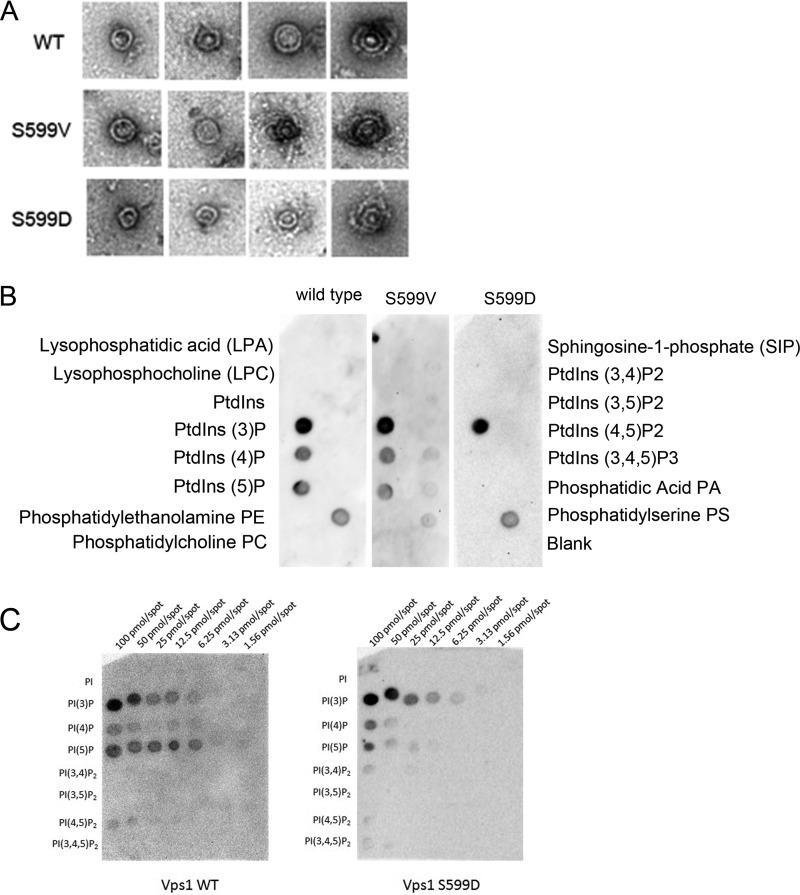
*In vitro* analysis of Vps1 and the S599 mutants. (A) Recombinant Vps1 was purified, and oligomers were visualized when samples were processed and stained as described in the text. Shown are examples of single rings (three images for each protein) and double-ring structures. (B) Lipid binding preferences were investigated using a PIP strip assay following incubation with purified proteins and detection with anti-His antibodies. (C) PIP arrays were performed using wild-type and S599D mutant proteins to determine whether differences between binding on the PIP strip were due to a complete block in binding caused by the mutation or whether there was a more subtle change in lipid affinity.

In order to gain further insight into the effect of Vps1 S599 mutants on protein function, mutant forms of the protein were purified. Recently, we were able to demonstrate that purified Vps1 protein can form oligomeric ring structures (38). The two Vps1 S599 mutants were purified and examined using transmission electron microscopy following negative staining. As shown in Fig. 7A, both mutants were able to form ring structures similar to those found with wild-type protein. The sizes of the uniform single rings were 32.2 ± 3.7 nm for wild-type Vps1 and 31.4 ± 2.9 nm and 30.5 ± 3.3 nm for the S599V and S599D mutants, respectively. The capacity to form higher-order, double-ring structures also seems to be maintained.

In previous work, we have also shown that Vps1 can bind liposomes, but we had not undertaken an analysis of lipid binding preferences (14). Using a dot blot approach with PIP strips, the wild-type and mutant proteins were incubated overnight to allow interaction, and binding was detected with anti-His antibodies. As shown in Fig. 7B, the wild-type Vps1 and the S599V mutant have very similar profiles of lipid binding, with clear binding only to the phosphatidylinositol monophosphates. The strongest binding appeared to be for phosphatidyl 3-phosphate [PI(3)P]. The S599D mutation did cause a reduction in binding to PI(4)P or PI(5)P but did not appear to affect PI(3)P binding. PIP arrays that carry different levels of lipids were then used to determine the extent of this apparent loss of PIP binding in the S599D mutant. The wild-type (nonphosphorylated recombinant) Vps1 and the phosphomimetic Vps1 S599D mutant proteins were incubated with the membranes before probing with anti-His antibodies. As shown in Fig. 7C, both wild-type Vps1 and Vps1 S599D were observed to bind most strongly to all phosphatidylinositol monophosphate lipids, with the strongest binding to PI(3)P and with very weak binding to PI(4,5)P2 detectable. This finding suggests that the S599D mutation causes a slight but not complete reduction in the ability of the protein to interact with PI(4) and PI(5)P. It also suggests that the ability of Vps1 to localize to membranes may be at least in part associated with lipid binding, as analysis of Vps1-GFP shows the strongest localization to endosomes that are enriched in PI(3)P (49, 50).

## DISCUSSION

In this work, we have demonstrated that the yeast dynamin Vps1 is phosphorylated on multiple residues. *In vivo*, it appears that the kinase Pho85 is important for the Ser599 phosphorylation event and that this modification regulates Vps1 interaction with the amphiphysin Rvs167. Mutation of the S599 site, however, does not affect the ability of Vps1 to be localized to endocytic sites. When phosphorylated, Vps1 can interact with Rvs167, but this is not sufficient for completing scission, as a delay in endocytic scission and elongated invaginations are observed in cells expressing the Vps1 S599D mutant. This suggests that dephosphorylation of Vps1 is required to complete the cycle and facilitate the scission step.

Rvs167 itself is phosphorylated by the kinase Pho85/Pcl2 on three serine-proline (SP) motifs in the region between its BAR domain and its SH3 domain (31). The functions of these modifications have not been explored in depth, but lack of phosphorylation of Rvs167 by Pho85 correlates with loss of Rvs167 SH3 domain binding to the yeast WASP homolog Las17 (31). Rvs167 has also been shown to bind directly to lipids and, in conjunction with Rvs161, to promote lipid tubule formation (51). The data we show here support our previous work showing the importance of an interaction between Rvs167 and Vps1 during endocytosis. The Vps1 S599V mutation completely abrogates the yeast two-hybrid interaction with Rvs167. Overexpression of the S599V mutant also failed to rescue the temperature sensitivity caused by the expression of excess Rvs167 in cells. In contrast, the S599D mutant can still interact with Rvs167 in a yeast two-hybrid assay. The interaction level appears slightly reduced, but this is possibly due to the aspartate residue not being a fully effective replacement for the phosphate group that would be present on the wild-type protein. S599D overexpression does, however, interact sufficiently well to rescue the Rvs167 overexpression phenotype. It is currently unclear whether Vps1 interacts with the Rvs161/Rvs167 amphiphysin heterodimer *in vivo* or whether it interacts with Rvs167 in a distinct interaction. While most data have pointed to a function of Rvs161 and Rvs167 as an obligate heterodimer, the reason for the higher number of Rvs167 molecules reported in cells and the differential stability when one or the other gene is deleted is currently unclear (52, 53).

There are a number of models that can be put forward to bring together the available data. The simplest of these is that Vps1 is recruited to endocytic sites just prior to invagination. The effect of some Vps1 mutants, causing an early defect in the ability of the membrane to invaginate rather than just affecting the postinvagination scission stage, supports this time of arrival (14, 38). Following recruitment, Vps1 assembles into rings and higher-order structures. We propose that it is in this form that the Pho85 kinase is able to phosphorylate Vps1 and thereby promote an interaction with Rvs167. This interaction might either support the recruitment of higher levels of Rvs167 or promote reorganization and oligomerization of Rvs167 at the site. Given that we have shown Vps1 can form oligomeric rings of a defined size (24), it is possible that Vps1 forms a template structure for amphiphysin BAR domain oligomerization and thereby promotes invagination. Alternatively, or in addition, the Vps1 rings might facilitate a curvature of the membrane that aids the membrane interaction of the amphiphysin itself.

Data on the ultrastructure of invaginations in the Vps1 S599D mutant indicate that the final scission step requires dephosphorylation. This would act to reduce the Vps1-Rvs167 interaction and allow scission to proceed. If Rvs167 and Vps1 were phosphorylated by Pho85 at the same stage, it would mean that during membrane invagination, Rvs167 would be phosphorylated and thus unable to bind Las17. However, following phosphatase action, as well as promoting disassembly of Vps1-Rvs167, this would also allow Rvs167 to interact with Las17. Such a mechanism could then be viewed as a route to bring Las17 and its inherent actin-nucleating capacity to the site of membrane fission (54). Coupled with the direct interaction between actin and dynamin, actin polymerization at the site of the invagination then either could directly drive scission or could be associated with subsequent movement of the released vesicle within the cytoplasm (24, 29). We have recently demonstrated that overexpression of an early endocytic protein, Ysc84, that binds Las17 at a site overlapping Rvs167 can cause retractions in endocytosis similar to the *rvs167* deletion, supporting the *in vivo* importance of the Rvs167-Las17 interaction (55).

In mammalian cells, it is clear that phosphorylation in the C-terminal PRD region of dynamin is important for interactions with a number of endocytic proteins, including the BAR domain proteins amphiphysin and syndapin (32, 34, 36, 56–58). Dynamin-1 has been shown to be maintained with high phosphorylation levels in resting cells, and membrane depolarization coupled with activation of the phosphatase calcineurin can drive rapid dephosphorylation and subsequent endocytosis. In the cases of Vps1 and Rvs167, the identity of the phosphatase is currently unknown. However, the essential type 1 phosphatase Glc7 has been shown to interact with, and be required for, dephosphorylation reactions of a number of proteins at the endocytic site (57, 59–63)

The S599 residue that we have studied here lies in the insert B region of Vps1. Our previous data showing a PXXP SH3 binding motif in insert B, and the fact that we observe liposome binding in the absence of a PH domain, has led us to propose that insert B might be able to confer both lipid binding and SH3 binding functions on this dynamin protein. The S599V mutation reduces the interaction with Rvs167 in cells, indicating the importance of the S599 phosphorylation for SH3 domain binding. There was also a subtle but reproducible effect detected on the PIP strips caused by the S599D mutation, suggesting that phosphorylation in the region might also modulate lipid binding. Given the identification of other phosphorylation events within this region (S570 and S579), these might, alone or together, cause further lipid or protein binding changes, and these possibilities will be the focus of future studies. It will also be of interest to determine whether the other phosphorylation events identified affect endocytosis or other distinct functions of the Vps1 protein. The more N-terminal phosphorylation sites identified lie in both the GTPase and the stalk domain. The threonine residues 242 and 247 in the GTPase domain were indicated to be high-confidence phosphorylation sites in mass spectrometry analysis. These residues are highly conserved (T200 and T205 in Dyn1) and may indicate that some regulatory sites could have been maintained throughout evolution. Interestingly, residue T205 is part of the dynamin G4 loop that plays a critical role in the G-G domain dimerization (64). Thus, phosphorylation at this site in yeast Vps1 and potentially in classical dynamins would likely have a significant effect on the ability of the protein to form oligomers.

Overall, our data indicate that phosphorylation is a key modification allowing Vps1 to make distinct protein interactions that facilitate the progression of endocytosis.
